# Genome-wide association and pathway analysis of feed efficiency in pigs reveal candidate genes and pathways for residual feed intake

**DOI:** 10.3389/fgene.2014.00307

**Published:** 2014-09-09

**Authors:** Duy N. Do, Anders B. Strathe, Tage Ostersen, Sameer D. Pant, Haja N. Kadarmideen

**Affiliations:** ^1^Section of Animal Genetics, Bioinformatics and Breeding, Department of Veterinary Clinical and Animal Sciences, Faculty of Health and Medical Sciences, University of CopenhagenFrederiksberg, Denmark; ^2^Pig Research Centre, Danish Agriculture and Food CouncilCopenhagen, Denmark

**Keywords:** GWAS, pigs, pathway analysis, residual feed intake

## Abstract

Residual feed intake (RFI) is a complex trait that is economically important for livestock production; however, the genetic and biological mechanisms regulating RFI are largely unknown in pigs. Therefore, the study aimed to identify single nucleotide polymorphisms (SNPs), candidate genes and biological pathways involved in regulating RFI using Genome-wide association (GWA) and pathway analyses. A total of 596 Yorkshire boars with phenotypes for two different measures of RFI (RFI1 and 2) and 60k genotypic data was used. GWA analysis was performed using a univariate mixed model and 12 and 7 SNPs were found to be significantly associated with RFI1 and RFI2, respectively. Several genes such as *xin actin-binding repeat-containing protein 2* (*XIRP2*),*tetratricopeptide repeat domain 29* (*TTC29*),*suppressor of glucose, autophagy associated 1* (*SOGA1*),*MAS1*,*G-protein-coupled receptor (GPCR) kinase 5* (*GRK5*),*prospero-homeobox protein 1* (*PROX1*),*GPCR 155* (*GPR155*), and *FYVE domain containing the 26* (*ZFYVE26*) were identified as putative candidates for RFI based on their genomic location in the vicinity of these SNPs. Genes located within 50 kbp of SNPs significantly associated with RFI and RFI2 (*q*-value ≤ 0.2) were subsequently used for pathway analyses. These analyses were performed by assigning genes to biological pathways and then testing the association of individual pathways with RFI using a Fisher’s exact test. Metabolic pathway was significantly associated with both RFIs. Other biological pathways regulating phagosome, tight junctions, olfactory transduction, and insulin secretion were significantly associated with both RFI traits when relaxed threshold for cut-off *p*-value was used (*p* ≤ 0.05). These results implied porcine RFI is regulated by multiple biological mechanisms, although the metabolic processes might be the most important. Olfactory transduction pathway controlling the perception of feed via smell, insulin pathway controlling food intake might be important pathways for RFI. Furthermore, our study revealed key genes and genetic variants that control feed efficiency that could potentially be useful for genetic selection of more feed efficient pigs.

## INTRODUCTION

Residual feed intake is defined as the difference between the observed feed intake and the feed intake predicted based on production traits such as average daily gain and backfat thickness. RFI is a sensitive and accurate indicator of feed efficiency in livestock that is being increasingly accepted as an alternative measure for feed efficiency in livestock species. Genetic selection for animals with reduced RFI can be advantageous from both economic and environmental perspectives (Dekkers and Gilbert, 2010; Cruzen et al., 2012; Saintilan et al., 2013). However, genetic variants and biological mechanisms regulating RFI need to be identified, which would help to improve genetic selection for this trait. GWA, a hypothesis-free approach that uses a large numbers of SNPs spread throughout the genome to identify quantitative trait loci (QTL) potentially harboring candidate loci, has been widely used to explore the genetics underlying complex traits. Past studies have led to the identification of many QTLs influencing feed conversion ratio (FCR) in pigs^1^. FCR is currently the only available measure of feed efficiency that is included in the selection index for the Danish pig breeds. However, ratio traits such as FCR are not ideal for statistical and biological reasons (Gunsett, 1984) and the accurate definition for feed efficiency in animals is still being debated. Recently, several studies have been conducted to identify QTLs and candidate genes putatively influencing RFI in pigs. Using a Piétrain–Large White backcross population, Gilbert et al. (2010) identified QTLs on pig chromosomes (SSC) 5 and 9 for RFI in growing pigs. In Yorkshire pigs, a GWA study revealed several significant SNPs on SSC 2, 3, 5, 7, 8, 9, 14, and 15 influencing RFI (Onteru et al., 2013). A candicate gene study performed by Fan et al. (2010) validated these SNPs in *FTO* and *TCF7L2* genes as genetic markers for RFI in pigs. Recently, Shirali et al. (2013) detected novel QTLs for residual energy intake on SSC 2, 4, 7, 8, and 14 in a crossed populations (Pietrain grand-sires crossed with grand-dams bred from a three-way cross of Leicoma boars with Landrace × Large White dams). Sanchez et al. (2014) detected a SNP on SSC 6 for RFI in Large White pigs.

Recently, we have identified significant SNPs on SSC 1, 9, and 13 for RFI in Duroc pigs (Do et al., 2014). Danish Durocs, used as terminal sires in combination with crossbred LY sows (Landrace × Yorkshire), are bred with a higher emphasis on growth and feed efficiency traits compared to Yorkshire pigs, where the emphasis is considerably more on improving litter size. Given these differing emphases on selection, it is reasonable that the genetic architeture of these two breeds differs with respect to traits like feed efficiency that are targetted more intesively for selection within Durocs. In accordance with this, we have found that the genetic variation (heritability) of RFI is higher in Yorkshire compared to Duroc pigs (Do et al., 2013a). Therefore, while the biological mechanisms are likely conserved even across species (Mayr, 1963; Raff, 1996), the genetic regulation of these mechanisms is not necessarily conserved, and investigating the genetics underlying the same phenotype in a different breed could provide novel insights into the biological mechanisms underlying feed efficiency. Comparing findings of genomic investigations on different breeds that have differing linkage disequilibrium (LD) structure could also potentially assist in narrowing the boundaries of putative QTL regions.

While GWA studies have been reasonably successful, they often focus on a top few significant SNPs while ignoring other SNPs with lower significance levels that could still be biologically relevant. Gene set enrichment and pathway analyses using publicly available biological databases could potentially complement efforts to identify causal loci for complex traits, as has been shown in previous studies (Kadarmideen, 2008; Torkamani et al., 2008; Wang et al., 2010). These approaches, instead of relying solely on statistically associated genetic variants, focus on biological pathways that are mediated by genes located in the vicinity of these variants. Such approaches have been shown to provide valuable insights into the biology underlying complex phenotypes (Kadarmideen et al., 2006; Farber, 2013; Kadarmideen, 2014). Therefore, the objective of our study was to use both GWA and pathway analyses to identify SNPs, genes, and biological pathways that could potentially influence RFI in Yorkshire pigs.

## MATERIALS AND METHODS

### ESTIMATION OF RESIDUAL FEED INTAKE AND DEREGRESSED ESTIMATED BREEDING VALUES

Data were recorded during a 5-year period (2008–2012) and supplied by the Pig Research Centre of the Danish Agriculture and Food Council. A total of 596 Yorkshire pigs had both phenotypic (RFI) and genotypic records (based on PorcineSNP60 Illumina iSelect BeadChip). The method of calculation of RFI has been previously discussed in detail (Do et al., 2013a). In summary, RFI was computed as the difference between the observed average daily feed intake and the predicted daily feed intake using two statistical models. In the first model (RFI1), predicted daily feed intake was estimated using linear regression of daily feed intake on initial test weight (BWd) and average daily gain from 30 to 100 kg, whereas in the second model (RFI2), backfat was used as an additional regressor. The EBVs for RFI were calculated using a univariate animal model where barn–year–season were used as fixed effects and the effect of pen and the additive genetic effect were treated as random effects. The pedigree was traced back to January, 1971 and included 14,681 pigs with 1951 sires, 6766 dams. These EBVs were further deregressed as previously described (Ostersen et al., 2011; Do et al., 2013b), following the deregression procedure of Garrick et al. (2009). This procedure adjusts for ancestral information, so that the deregressed EBV (dEBVs) only includes information from individual animals and their descendants. Since our resource population consists of 5337 pigs of which only 1564 pigs had genotypic records, the use of deregressed proofs was intended to maximize use of phenotypic information from non-genotyped pigs. Because the dEBVs have unequal variances, they should be used in a weighted analysis. The weight for the *i*th animal was estimated as:

$$w_{i}=\frac{\left(1:h^{2}\right)}{\left[\left(c+\left(\left(1-r_{i}^{2}\right)/r_{i}^{2}\right)\right)\,h^{2}\right]}$$

in which *c* was the part of the genetic variance that was assumed to be not explained by markers (*c* = 0.1), *h*^2^ was the heritability of the trait, and $r_{i}^{2}$ was the reliability of the dEBV of the *i*th animal.

### GENOTYPING AND DATA QUALITY CONTROL

The details of the resource population used and DNA collection were described in Henryon et al. (2001). For genotyping, genomic DNA was isolated from tissue by treatment with proteinase K followed by sodium chloride precipitation and SNPs were genotyped on the PorcineSNP60 Illumina iSelect BeadChip. Data quality control prior to GWA analyses was implemented by discarding animals and SNPs with a call rate <0.95, SNPs deviating from Hardy Weinberg equilibrium (*p* < 0.0001) and SNPs with a MAF < 0.05.

### LINEAR MIXED MODEL USED FOR GENOME WIDE ASSOCIATION ANALYSES

A univariate linear mixed model was implemented to test the association between each SNP and RFI. The model was similar with previous GWA analysis in Duroc pigs (Do et al., 2014). In summary, the model for each SNP (analyzed individually) was as follows:

y=1⁢μ+Z⁢a+m⁢g+e

where *y* is the vector of dEBVs for RFI, 1is a vector of 1s with length equal to number of observations, *μ* is the general mean, *Z* is an incidence matrix relating phenotypes to the corresponding random polygenic effect, *a* is a vector of the random polygenic effect ∼N(0,A$\sigma_{u}^{2}$) where A is the additive relationship matrix and $\sigma_{u}^{2}$ is the polygenic variance, m is a vector with genotypic indicators (-1, 0, or 1) associating records to the marker effect, *g* is a scalar of the associated additive effect of the SNP, and *e* is a vector of random environmental deviates: N(0,W^-1^$\sigma_{e}^{2}$) where $\sigma_{e}^{2}$ is the general error variance and *W* is the diagonal matrix containing weights of the dEBVs. The model was fitted by restricted maximum likelihood (REML) using the DMU software (Madsen et al., 2006) and testing was done using a Wald test against a null hypothesis of *g* = 0. The Wald test was based on a *t*-distribution and regression coefficients and SEs were obtained by solving linear mixed model equations using DMU (Madsen et al., 2006). This test was done by the *t*-distribution function “pt()” in R with *p* = 2^∗^pt[abs(β/*SE*), (*n* - 3), log = FALSE, lower.tail = FALSE; where β is regression coefficient, *SE* is standard error estimated based on the inverse of the mixed model coefficient matrix and *n* is number of SNPs in the genotype data]. Bonferroni corrected significance threshold, used to account for multiple comparisons, was estimated at a *p* = 1.31e-06. However, Bonferroni correction is known to be overly conservative especially when genetic data exhibits high LD, which could produce false negative results (Duggal et al., 2008). Therefore, in our analyses we considered a less conservative significance threshold of a *p* = 1e-04 in order to account for multiple tests. The *p* was chosen here based on a Bonferroni adjustment only for the number of independent tests that was in turn inferred by the number of principal components accounting for a 99% of the variance of the SNP matrix (Gao et al., 2008). Moreover, to further characterize candidate regions affecting RFI, we performed LD block analyses for the chromosomal regions with multiple (or the most) significant SNPs clustered. The blocks were defined based on criteria suggested by Gabriel et al. (2002) which implemented in Haploview (Barrett et al., 2005).

### PATHWAY ANALYSES

#### Assignment of genes to SNPs

Assigning genes close to a few SNPs with high statistical significance, and ignoring many SNPs with lower significance levels could result in missing out on key putative candidates and associated pathways. Hence, we used the procedure of controlling false discovery rate (FDR; Benjamini and Hochberg, 1995) to select SNPs for pathways analyses. All SNPs with a FDR (or *q*-value) ≤0.2 were used to identify putative candidate genes. Based on previous studies, we also included pathway annotations associated with genes within 50 kb of SNPs associated with RFI at a nominal significance thresold of 0.05, in pathway analyses (Choquette et al., 2012; Peñagaricano et al., 2013). Positional candidate genes, located within 50 kb of these SNPs, were identified using function GetNeighGenes() in the NCBI2R package^2^ for R program (R Development Core Team, 2008). The distance of 50 kb was used in order to capture proximal regulatory and other functional regions close to the gene. Moreover, several studies showed that the average LD was high in pigs. The average distance between adjacent SNP pairs (with average *r*^2^ = 0.5) was around 72 kb in the current population (Wang et al., 2013). Therefore, the distance of 50 kb was suitable to capture the causal genes/SNPs. Individually, each gene was considered to be significantly associated with RFI if a SNP with a *q*-value ≤0.2 (as well as for relxed thresold at a *p* ≤ 0.05) was located either inside the genic region or within 50 kb of the genes.

#### Assignment of genes to Kyoto Encyclopedia of Genes and Genomes (KEGG) pathways and pathway analyses

For functional annotation, Kyoto Encyclopedia of Genes and Genomes^3^ (KEGG) was used for getting pathways. To assign genes to pathways, we used the function GetPathways() in NCBI2R package; and to get number and names of genes in each pathway, the mapPathwaytoname() function^4^ was used. Because RFI was production trait in pigs, the assigned pathways belonging to human diseases and drug development categories were removed. To determine whether a pathway term was significantly associated with RFI, we tested if genes significantly associated with RFI were overrepresented amongst all the genes of any given pathway. This association analysis was performed using a Fisher’s exact test via the fisher.test() function in R.

## RESULTS

### GENOME-WIDE ASSOCIATION ANALYSES

**FIGURE 1:**
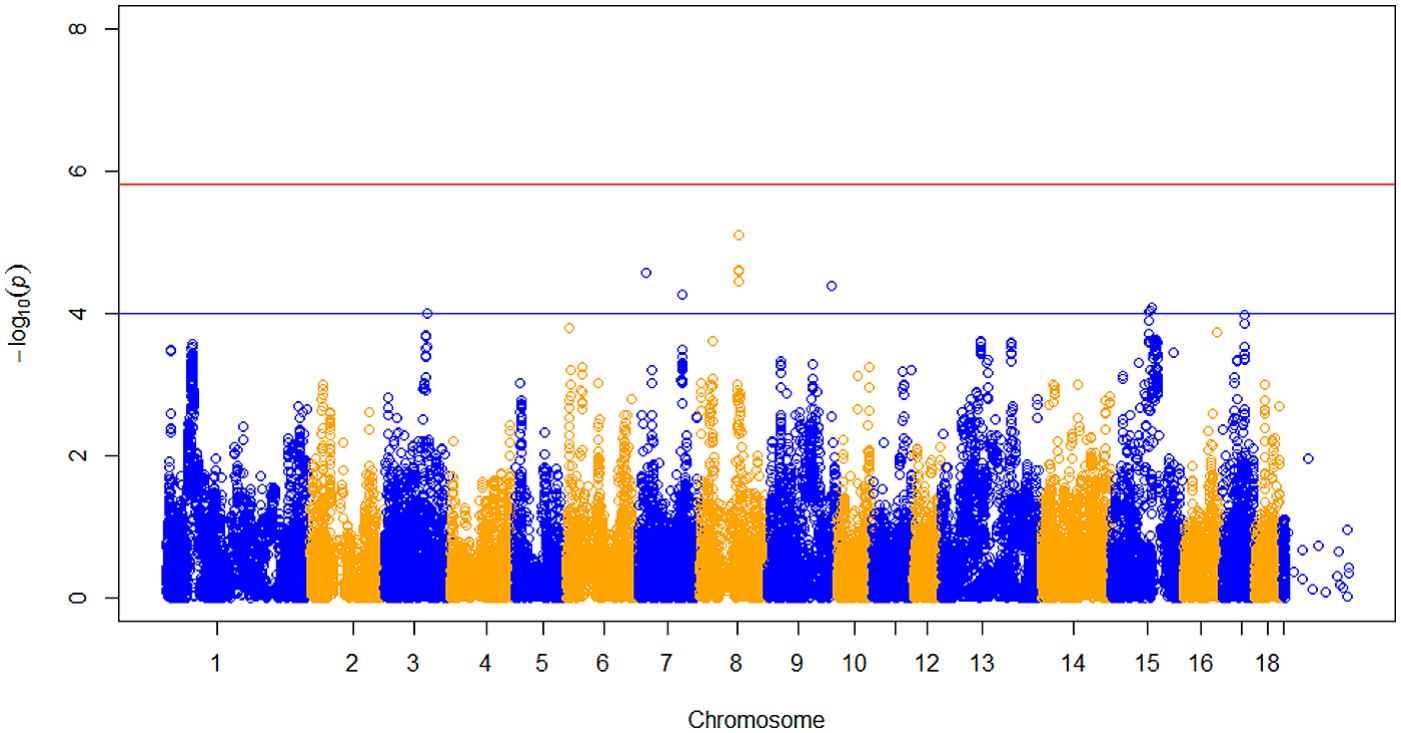
**Manhattan plot of genome-wide *p* of association for residual feed intake 1 (RFI).** The horizontal red and blue line represents the Bonferroni (*p* = 1.31e-06) and genome-wide significance threshold (*p* ≤ 1e-04), respectively.

**FIGURE 2:**
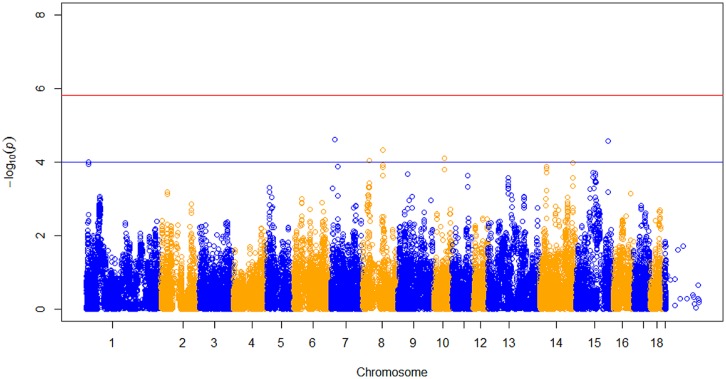
**Manhattan plot of genome-wide *p* of association for RFI 2.** The horizontal red and blue line represents the Bonferroni (*p* = 1.31e-06) and genome-wide significance threshold (*p* ≤ 1e-04), respectively.

**FIGURE 3:**
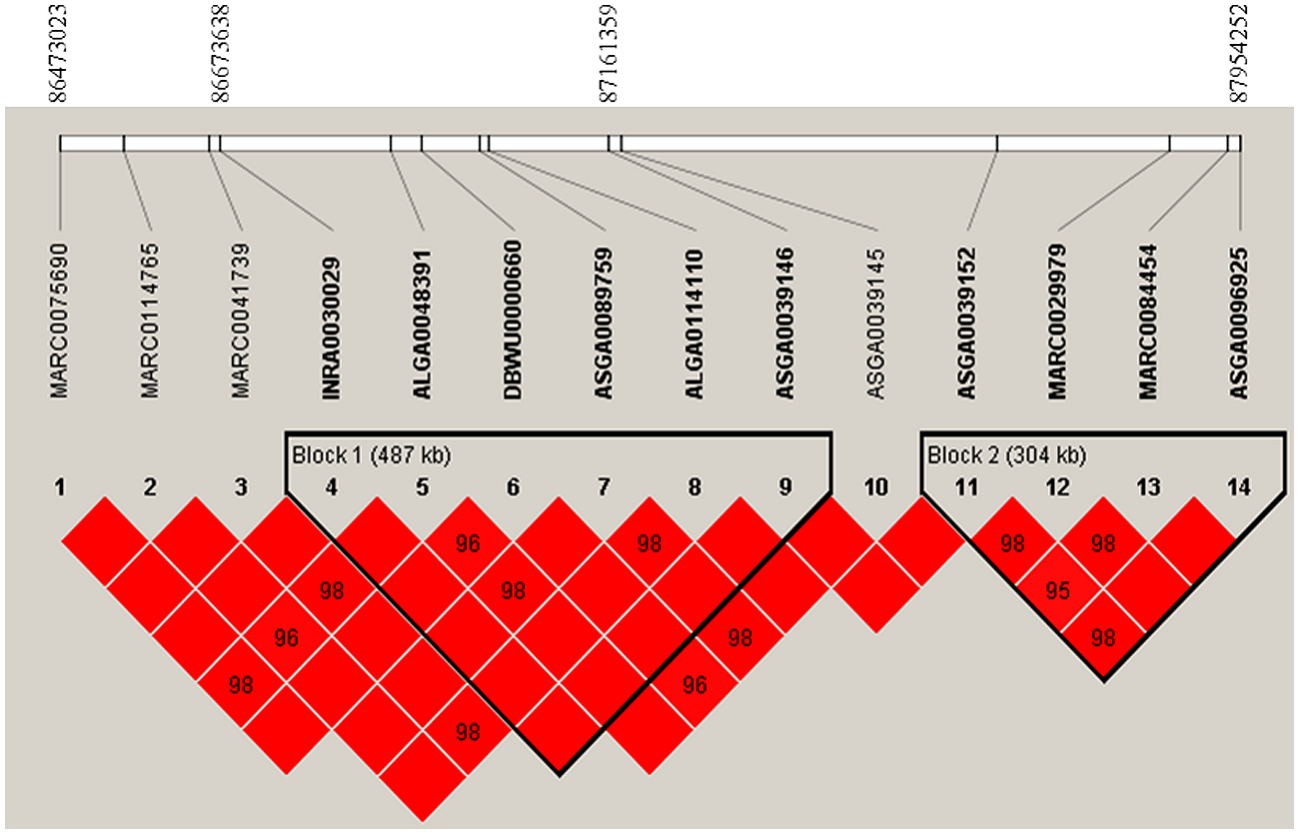
**Linkage disequilibrium (LD) pattern on a region from 86 to 88 Mb on pig chromosome 8.** LD blocks are marked with triangles. Values in boxes are LD (*r^2^*) between SNP pairs and the boxes are red indicated LOD > 2 and *D*′ = 1 (LOD is the log of the likelihood odds ratio, a measure of confidence in the value of *D*′).

After data quality control, a total of 37,192 SNPs and 596 pigs remained in the final dataset for GWA analyses. Eleven SNPs were significantly associated with RFI1 (**Figure 1**), and seven were associated with RFI2 (**Figure 2**); two SNPs (MARC0027992 and ASGA0039145) being associated with both traits [*p* ≤ 1e-04 (0.0001)]. The most significant associations were found between ASGA0039145 and RFI1 (*p* = 7.76e-06) and between MARC0027992 and RFI2 (*p* = 2.47e-05). Significant SNPs associated with RFI1 were located on SSC 3, 7, 8, 9, 15, and 17 meanwhile those for RFI2 were located in SSC 1, 7, 8, 10, 14, and 15 (**Table 1**). A search for genes located in the immediate 50 kbp vicinity of these SNPs revealed *XIRP2*,*TTC29*,*SOGA1*,*MAS1*,*G*RK5,*PROX1*,*GPR155*, and *ZFYVE26* as putative candidates. Two LD blocks were detected on a candidate region (86–88 Mb on SSC8) which associated with both RFI (**Figure 3**).

**Table 1:** Genome-wide significant SNPs for residual feed intake in Yorkshire.

Trait^1^	SNP	SSC^2^	Positions	*P*	Nearest gene	Gene positions^3^	Gene names
RFI1	ALGA0085846	15	82,835,779	9.7e-05	*XIRP2*	15:82,491,704-82,597,617	Xin actin-binding repeat-containing protein 2
RFI1	ALGA0085859	15	83,426,312	9.1e-05	*ENSSSCG00000023421*	15:83,412,046-83,526,943	Novel gene
RFI1	ALGA0114110	8	87,012,660	3.6e-05	*TTC29*	8:86,904,297-86,936,988	Tetratricopeptide Repeat Domain 29
RFI1	ASGA0039145	8	87,177,392	7.8e-06	*ENSSSCG00000009034*	8:87,200,641-87,201,588	Novel gene
RFI1	ASGA0039146	8	87,161,359	2.5e-05	*ENSSSCG00000009034*	8:87,200,641-87,201,588	Novel gene
RFI1	ASGA0089759	8	87,000,474	2.6e-05	*TTC29*	8:86,904,297-86,936,988	Tetratricopeptide Repeat Domain 29
RFI1	ASGA0103232	9	142,526,652	4.2e-05	*PROX1*	9:142,428,903-142,469,651	Prospero homeobox protein 1
RFI1	MARC0027992	7	18,810,414	2.7e-05	*ENSSSCG00000018237*	7:18,702,099-18,702,261	U1 spliceosomal RNA
RFI1	MARC0082350	15	89,623,572	8.4e-05	*GPR155*	15:89,587,688-89,695,502	G protein-coupled receptor 155
RFI1	ALGA0043644	7	97,889,360	5.4e-05	*ZFYVE26*	7:97,825,802-97,898,510	Zinc finger, FYVE domain containing 26
RFI1	H3GA0010038	3	94,756,601	1.0e-04	*ENSSSCG00000008418*	3: 95,389,252-95,546,928	Novel gene
RFI1	MARC0067053	17	45,539,648	1.1e-04	*SOGA1*	17: 45,467,693-45,539,349	Suppressor of glucose, autophagy associated 1
RFI2	ALGA0117721	10	44,751,617	8.1e-05	*ENSSSCG00000011017*	10:44,914,173-44,928,565	Novel gene
RFI2	ASGA0039145	8	87,177,392	4.7e-05	*ENSSSCG00000009034*	8:87,200,641-87,201,588	Novel gene
RFI2	DRGA0008464	8	27,798,101	9.0e-05	*RPS18*	8:28,015,649-28,016,107	Ribosomal protein S18
RFI2	H3GA0045110	15	136,035,441	2.7e-05	*ENSSSCG00000026566*	15:135,772,108-135,772,198	Novel microRNA
RFI2	MARC0027992	7	18,810,414	2.5e-05	*ENSSSCG00000018237*	7:18,702,099-18,702,261	U1 spliceosomal RNA
RFI2	MARC0072970	1	9,324,586	1.0e-04	*MAS1*	1: 9,326,800-9,327,777	MAS1 oncogene
RFI2	H3GA0042665	14	140,742,709	1.0e-04	*GRK5*	14: 140,637,968-140,875,398	G protein-coupled receptor kinase 5

*^1^RFI1: residual feed intake 1; RFI2: residual feed intake 2.*

*^2^Pig chromosome.*

*^3^Based on Sscrofa10.2 (GCA_000003025.4) at http://www.ensembl.org/Sus_scrofa/Info/Index*

### PATHWAY-BASED ASSOCIATION ANALYSES

Based on results from GWA analyses, a total of 402 SNPs associated with RFI1 and another 304 SNPs associated with RFI2 (based on a FDR threshold of *q*-value ≤0.2) were used to locate 339 and 304 genes, respectively, within 50 kb of these SNPs for pathway analyses. A total of 21,296 genes in 50 kb flanking regions of SNPs which passed QC was used as background for enrichment test. Pathway analysis tests for KEGG pathways revealed a metabolic pathway to be associated with both RFI1 (*p* = 0.008) and RFI2 (*p* = 0.002); and an additional olfactory transduction pathway only associated with RFI2 (*p* = 0.03). Repeating pathway analyses after relaxing the significance threshold to *p* ≤ 0.05, revealed 15 and 12 pathways associated with RFI1 and RFI2, respectively (Table S1 in Supplementary Materials), of which nine pathways were commonly associated with both RFI phenotypes. The pathways associated with both RFI phenotypes included metabolic pathway, olfactory transduction, tight junction and phagosome pathway that were associated with both RFI traits at *p* ≤ 0.01.

## DISCUSSION

The statistical and bioinformatics methods used in the current study are similar to those applied in a separate study aiming to identify QTLs influencing RFI in Duroc pigs (Do et al., 2014). However, in order to account for multiple comparisons, we used a less stringent genome-wide significance threshold (*p* ≤ 0.0001) that adjusts the nominal significance threshold only for the number of independent tests that are possible given a particular dataset. The Bonferroni correction that was used in the previous study (Do et al., 2014), is known to overcompensate for multiple testing, especially when applied to correlated data. As such, the use of a relaxed threshold was expected to decrease the number of false negatives thereby increasing power. Several studies have applied pathway analysis on GWA datasets and reported pathways and GOs associated with backfat thickness (Fontanesi et al., 2012), feeding behavior (Do et al., 2013b), and RFI (Do et al., 2014) in pigs. However, these studies focused on genes in close proximity to significantly associated SNPs based on stringent genome-wide thresholds like Bonferroni, while ignoring many SNPs with lower significance levels. Therefore, we used a lower significance threshold including all genes located near SNPs associated with RFI at a *q*-value ≤0.2 in the current study. (Peñagaricano et al., 2013) also used more relaxed threshold with *p* ≤ 0.05 and detected many significant pathways and network from GWA data for bull fertility traits. Moreover, since the porcine genome contains many uncharacterized genes, widely used annotation servers like DAVID^5^ and GOEAST^6^ are of limited use, as they perform poorly in converting porcine gene IDs. Therefore, we performed functional annotation of genes by directly querying KEGG databases that are better able to handle porcine gene IDs.

Comparing results from our previous study targeting a Duroc resource population almost twice in size, we were able to identify overlapping QTLs at approximately 87 Mb on SSC 8, and at approximately 136 Mb on SSC 15 (**Table 1**; Do et al., 2014). The proportionally small number of overlapping QTLs is in agreement with other studies that have investigated QTLs for a specific trait within different pig breeds (Gregersen et al., 2012). Furthermore, despite of substantial differences in the location of QTL regions between the two breeds, pathway analyses identified many pathways (e.g., insulin regulation related pathways and cellular communication pathways) that were also identified in our previous study with Duroc. Taken together, these observations reaffirm the notion that while the biological mechanisms underlying a particular phenotype do not differ, the genetic regulation of these mechanisms can differ between breeds. This is particularly important in the context of developing marker-assisted and genomic selection strategies, as it demonstrates that improving the same trait may require different sets of markers for different lines/breeds of livestock.

### CANDIDATE GENES FOR RESIDUAL FEED INTAKE

Significant associations between both RFI and SNPs on SSC 7 (MARC0027992) and 8 (ALGA0039145) were found in the present study. Here, *ENSSSCG00000018237* encoding U1 spliceosomal RNA was found near a SNP significantly associated with both RFIs on SSC 7. U1 spliceosomal RNA constitutes U1 small nuclear ribonucleoprotein that plays a role in splicing of pre-mRNAs (Zwieb, 1996). Another independent study (Onteru et al., 2013) reported a QTL region between 16 and 17 Mb on SSC 7 in Yorkshire pigs for RFI. In this study, a SNP significantly associated with RFI1 was found within an intron 4–5 of zinc finger, FYVE domain containing the 26 (*ZFYVE26*) gene that is also located on SSC 7. The gene encodes a protein containing a FYVE zinc finger binding domain which helps target the protein to membrane lipids (Laity et al., 2001). While *ZFYVE26* has been associated with autosomal recessive spastic paraplegia in humans (Herd et al., 2004), the precise biological function of this gene has not yet been described.

Another important association was between SNP ASGA0039145 on SSC 8 and both RFI traits. This SNP is located in a genomic region where QTLs influencing FCR have been reported in an independent study based on a Duroc resource population (Sahana et al., 2013). The *ENSSSCG00000009034* is a gene closest to this SNP; however, the gene has not been functionally characterized yet. Moreover, this SNP is tightly linked with five other SNPs to form the LD block 1 (**Figure 3**). This LD block spans 487 kb region and consists three significant SNPs for RFI1. This LD block also covers a *TTC29* gene which encodes a testis development protein that could also be an interesting candidate for further investigation. Also known as *NYD-SP14*, *TTC29* is a component of axonemal dyneins (Yamamoto et al., 2008) that have recently been demonstrated to play an important role in fat metabolism (Sohle et al., 2012).

We also found many SNPs to be associated with either RFI1 or RFI2 in our analyses. On SSC3, the SNP H3GA0010038 associated with only RFI1 located closest to a novel gene. On SSC 9, another transcriptional factor *PROX1* was found proximal to an SNP exclusively associated with RFI1. *PROX1* likely plays a fundamental role in the early development of the central nervous system (Kaltezioti et al., 2010). It also is a key regulator of lymphatic endothelial cell fate specification (Ma, 2007), ERRα mediated control of the molecular clock (Choquette et al., 2012), and modulation of insulin sensitivity and glucose handling (Fontanesi et al., 2012) that could influence energy metabolism and RFI. On SSC17, MARC0067053 is significantly associated with RFI1 (*p* = 1.08e-04), and is located in 5UTR′ region of *SOGA1* gene. This gene encodes a SOGA1 protein that plays a role in reducing glucose production (Cowerd et al., 2010). It also contributes to adiponectin-mediated insulin-dependent inhibition of autophagy (Forbes, 2010). Since autophagy provides biochemical intermediates for glucose production (Forbes, 2010) which influences feed consumption, SOGA1 could be an interesting candidate gene for RFI1.

Moreover, on SSC 1 and SSC 14, close to significant SNPs, we reported two members of G-protein couple receptor (*MAS1* and *GRK5*) as possible candidate genes for RFI2. For instance, the GRK5 regulates the GPCR signaling pathway and GRK5 deficiency led to insulin resistance and hepatic steatosis, or decreases diet-induced obesity and adipogenesis in mice (Wang et al., 2012b). On chromosome 10, a functionally uncharacterized gene *ENSSSCG00000011017* encoding a lysozyme-like ortholog, was located near ALGA0117721 that was significantly associated with RFI2. Other candidate genes in proximity to SNP associated exclusively with RFI1 or RFI2 were *RPS18*,*GPR155*, and*XIRP2.* Dysregulation of GPCR 155 (*GPR155*) is associated with higher feed efficiency in chicken while *RPS18*, encoding the 40S ribosomal protein S18, is involved in regulation of development (Laity et al., 2001). However, very little is known about the biological function of these genes and their relevance in the context of RFI is not apparent.

Notably, melanocortin 4 receptor (*MC4R*; SSC 1: 178,553,488-178,555,219) is perhaps most well-known candidate gene for feed efficiency or/and feed intake in pigs (Kim et al., 2000; Houston et al., 2004; Burgos et al., 2006; Davoli et al., 2012; Onteru et al., 2013). The MC4R gene codes for a G protein transmembrane receptor playing an important role in energy homeostasis control. In pigs, a SNP (missense substitution 298 Asp > Asn) in *MC4R* gene has been identified and associated to average daily gain, feed intake and fatness traits in many difference studies (Kim et al., 2000; Houston et al., 2004; Fan et al., 2009; Davoli et al., 2012). Moreover, Leptin (SSC 18: 21, 201, 786-21, 204, 671) plays a key role in regulating energy intake and expenditure and is a candidate gene for feed efficiency in pigs (Barb et al., 2001). However, variants in these genes are not included the PorcineSNP60 Illumina iSelect BeadChip and it is unclear whether such variants could influence RFI in the current population.

### STATISTICAL METHODS USED IN GWAS

One of the challenges for doing GWAS in livestock population is the large proportion of animals have phenotypic but no genotypic records, especially in dairy cattle. Recently, Wang et al. (2012a) proposed a single step GBLUP (or ssGBLUP) method that incorporates all genotypes, observed phenotypes and pedigree information jointly in one step and provides GEBVs for all animals with or without genomic data or phenotypic data or both (based on the methods of Aguilar et al., 2010; Christensen and Lund, 2010). The ssGWAS is a method based on GBLUP (Wang et al., 2014) which derives the SNP effects (or SNP variance) from GEBVs calculated from ssGBLUP. However, the use of the ssGWAS method is limited by finding appropriate number of iterations required to get marker solutions and most importantly, its inability to determine the genome-wide significance level for each SNP in the entire genomic data. However, our approach in this study of combining GWAS with pathways analysis requires a genome-wide adjusted *p*-value for each SNP for selecting the top SNPs for further downstream gene enrichment and pathway analyses using bioinformatics tools. Hence the genome-wide *p*-values for each SNP are not possible in ssGWAS method, we have adapted a mixed model GWAS and implemented using DMU package (Madsen et al., 2006). We used deregressed EBVs as a pseudo-phenotype but ssGBLUP would have also been a better choice in that it handles variability far better than the use of deregressed EBV as Wang et al. (2012a) reported differences in accuracy of genomic breeding values compared to other methods including classical GWAS.

There is still some controversy as to how to properly determine SEs of estimated SNP effects by GBLUP based-methods. Recently, Gualdron Duarte et al. (2014) provided a way to determine significance values for each SNP marker effect by linear transformations of genomic evaluations. Briefly, the likelihood ratio is calculated to test the significance of the largest effect segment of each chromosome by comparing against a reduced model with fixed effects and GEBVs. The critical value (size of the test) is adjusted by the Bonferroni correction. Moreover, we also would like to note that our GWA model could be extended to the case where the additive genetic relationship is substituted by the genomic relationship matrix like in EMMAX^7^.

### PATHWAYS INVOLVED IN RESIDUAL FEED INTAKE

Results from gene-set enrichment analyses are largely dependent on how gene-sets are identified or defined. In the current study, our gene-set was determined by the significance threshold that was used to declare SNPs significantly associated with RFI. Consequently, our enrichment analyses was very dependent on our choice of significance threshold. The choice of significance threshold also influences the degree of confidence that can be ascribed to results from gene-set enrichment analyses. Choosing a stringent threshold like Bonferroni will likely yield very few results with higher confidence as opposed to a lenient threshold that will likely yield more results with lower confidence. In our analyses, we decided to use a FDR based *q*-value threshold of 0.2 to balance the number of results and the degree of confidence associated with them. Applying more stringent FDR thresholds (for e.g., of 0.05 or 0.10) significantly reduced the numbers of SNP, and consequently the number of genes in the gene-set, for pathway annotation. Therefore, by setting the at a *q*-value ≤0.2 (*p* ∼ 0.001; meaning 20% of SNPs using for pathway analyses are likely to be false), we had a reasonable number of SNPs for gene-set enrichment analyses.

Regardless to different thresholds, the metabolic pathway^8^ was significantly associated with both RFI traits. Many previous studies have shown that variation in mediators of metabolic processes contribute to the variation of RFI (e.g., reviewed in Herd et al., 2004; Herd and Arthur, 2009; Hoque and Suzuki, 2009; Dekkers and Gilbert, 2010). However, the metabolic pathway is a broad overarching term that contains many specific modules (e.g., energy, carbohydrate and lipid, nucleotide and amino acid and secondary metabolism). So future investigations evaluating the contribution of specific sub-modules within this pathway to the genetic variation in RFI might be warranted. An interesting pathway associated with RFI2 (and with RFI1 when analysis is performed based on a nominal threshold of a *p* ≤ 0.05) was related to olfactory transduction. Olfactory transduction pathways are responsible for the perception of odor via olfactory receptors and downstream biochemical signaling events that ultimately get transformed into electrical impulses sent to the brain (Ma, 2007). Pigs have the largest repertoire of functional olfactory receptors (Groenen et al., 2012) encoded by at least 1113 genes (Nguyen et al., 2012), 14 and 11 of which are located near SNPs significantly associated with RFI1 and RFI2, respectively, (Table S1 in Supplementary Materials). Olfaction is one of the major sensory modalities that contribute to hedonic evaluation of a food, resulting in food choice and its possible consumption. It is modulated in response to changing levels of various molecules, such as ghrelin, orexins, neuropeptide Y, insulin, leptin, and cholecystokinin (Palouzier-Paulignan et al., 2012). These molecules are known to play an important role in controlling of RFI. For example, genetic selection for low and high RFI in pigs has been shown to change leptin concentration in plasma (Lefaucheur et al., 2011). Lower RFI has also been shown to be associated with lower serum leptin concentrations in Duroc pigs (Hoque et al., 2009).

Another interesting pathway significantly associated with both RFI traits (when using a nominal threshold of a *p* < 0.05) was the insulin secretion pathway (Table S1 in Supplementary Materials). Do et al. (2014) also reported Insulin signaling pathway associated with RFI in Duroc pigs. Insulin-dependent regulation of feed intake has been described in many species including cattle (Chen et al., 2012; Rolf et al., 2012) and pigs (Colditz, 2004; Cruzen et al., 2012). The results imply that insulin secretion is possibly an intermediary pathway by which olfactory transduction influences RFI. Richardson and Herd (2004) indicated that differences in some plasma metabolites and hormones have been positively related to genetic and phenotypic measures of RFI in ruminants.

The genetic variants identified by GWA studies may facilitate the incorporation of marker-assisted selection in commercial breeding schemes for improvement of complex traits. Moreover, Snelling et al. (2013) and Kadarmideen (2014) have suggested that genomic selection could perform better if it is guided by network and pathway analysis. Biological pathways identified by post-GWA analyses could further our current understanding of the genetic underlying different complex traits. Therefore, our results would be of interest not only to breeders interested in using marker-assisted selection to improve feed efficiency in pigs, but also to biologists interested in better understanding the biological mechanisms influencing feed efficiency. However, it is also important to consider potential limitations of our study, such as the limited size of Yorkshire resource population, statistical model used in the estimation of RFI, and statistical models used in GWA; gene set enrichment and pathway analyses. Finally, it is also important to note that all results reported in this study are only relevant to the specific definition used in this study.

In summary, the present study describes SNPs, candidate genes and biological pathways putatively influencing RFI in Yorkshire pigs. Important candidate genes such as *XIRP2*,*TTC29*,*SOGA1*,*MAS1*,*GRK5*,*PROX1*,*GPR155*, and *ZFYVE26* were identified here that could be further investigated for harboring causal variants. Pathway analyses identified metabolic and olfactory transduction pathways to be associated with RFI. Many other pathways (such as insulin secretion, tight junction) that were found to be associated with RFI based on a lenient nominal significance threshold might be of some interest. However, more studies are required to determine their role in regulating RFI.

## AUTHOR CONTRIBUTIONS

Duy N. Do did the analysis with the help of Haja N. Kadarmideen, Anders B. Strathe, Tage Ostersen, and Sameer D. Pant. Duy N. Do wrote the first draft of the manuscript. Haja N. Kadarmideen conceived and designed this project and provided supervision for Duy N. Do in all aspects of this research, including GWAS and biological interpretations. All authors contributed to writing of this manuscript, read and approved the final manuscript.

## Conflict of Interest Statement

The authors declare that the research was conducted in the absence of any commercial or financial relationships that could be construed as a potential conflict of interest.

## Footnotes

1 http://www.animalgenome.org/cgi-bin/QTLdb/SS/index

2 http://www.cran.r-project.org/web/packages/NCBI2R/index.html

3 http://www.genome.jp/kegg/

4 http://biobeat.wordpress.com/category/r/

5 http://david.abcc.ncifcrf.gov/

6 http://omicslab.genetics.ac.cn/GOEAST/

7 http://genetics.cs.ucla.edu/emmax/

8 http://www.genome.jp/kegg-bin/show_pathway?org_name=ssc&mapno=01100

## ABBREVIATIONS

Bp: base pairs
dEBV: deregressed estimated breeding values
EBV: estimated breeding values
FDR: false discovery rate
GWA: genome-wide association
MAF: minor allele frequency
Mb: mega base pairs
QTL: quantitative trait locus
RFI: residual feed intake
SNP: single nucleotide polymorphism.
