# Supplementary material for: Genome-wide association and pathway analysis of feed efficiency in pigs reveal candidate genes and pathways for residual feed intake
Source: Front Genet. 2014 Sep 9;5:307. doi: 10.3389/fgene.2014.00307 (PMC4159030; doi:10.3389/fgene.2014.00307)
Supplement: Supplementary file 1 [file Table_1.DOCX]

**Table S1.** Pathways involved in metabolism of residual feed intake (with relaxed threshold (p ≤ 0.05) was used)

| Trait^1^ | KEGG-PATHWAY | Number of genes in gene set | Number of genes in background | p |
| --- | --- | --- | --- | --- |
| RFI1 | Antigen processing and presentation | 13 | 44 | 1.06e-02 |
| RFI1 | Cell adhesion molecules (CAMs) | 24 | 95 | 1.59e-03 |
| RFI1 | ECM-receptor interaction | 16 | 58 | 5.20e-03 |
| RFI1 | Glycolysis / Gluconeogenesis | 13 | 44 | 1.06e-02 |
| RFI1 | GnRH signaling pathway | 14 | 58 | 2.63e-02 |
| RFI1 | Insulin secretion | 19 | 65 | 1.26e-03 |
| RFI1 | MAPK signaling pathway | 29 | 155 | 1.80e-02 |
| RFI1 | Metabolic pathway | 132 | 752 | 2.22e-06 |
| RFI1 | Olfactory transduction | 14 | 272 | 1.31e-03 |
| RFI1 | p53 signaling pathway | 11 | 41 | 3.09e-02 |
| RFI1 | Pancreatic secretion | 18 | 67 | 4.81e-03 |
| RFI1 | Phagosome | 25 | 94 | 5.47e-04 |
| RFI1 | Regulation of actin cytoskeleton | 23 | 124 | 4.97e-02 |
| RFI1 | Retrograde endocannabinoid signaling | 14 | 59 | 2.82e-02 |
| RFI1 | Tight junction | 20 | 84 | 7.23e-03 |
| RFI2 | Adherens junction | 12 | 50 | 4.43e-02 |
| RFI2 | ECM-receptor interaction | 13 | 58 | 3.94e-02 |
| RFI2 | Focal adhesion | 24 | 134 | 3.92e-02 |
| RFI2 | Glycolysis / Gluconeogenesis | 12 | 43 | 1.62e-02 |
| RFI2 | Insulin secretion | 16 | 65 | 1.11e-02 |
| RFI2 | MAPK signaling pathway | 27 | 155 | 3.07e-02 |
| RFI2 | Metabolic pathway | 115 | 751 | 3.83e-04 |
| RFI2 | Olfactory transduction | 11 | 272 | 2.04e-04 |
| RFI2 | Phagosome | 18 | 94 | 4.96e-02 |
| RFI2 | PI3K-Akt signaling pathway | 39 | 198 | 1.62e-03 |
| RFI2 | Regulation of actin cytoskeleton | 25 | 124 | 1.05e-02 |
| RFI2 | Tight junction | 21 | 84 | 2.13e-03 |

^1^: RFI1: residual feed intake 1, RFI2: residual feed intake 2
